# Fractional amplitude of low‐frequency fluctuation changes of specific cerebral regions in patients with toothache: A functional magnetic resonance imaging study

**DOI:** 10.1002/brb3.2937

**Published:** 2023-03-19

**Authors:** Jun Yang, Wan‐Xin Zeng, Jun Cheng, Min Kang, Xu‐Lin Liao, Ping Ying, Qian Ling, Jie Zou, Hong Wei, Yi‐Xin Wang, Ting Su, Yi Shao

**Affiliations:** ^1^ The Affiliated Stomatological Hospital of Nanchang University The Key Laboratory of Oral Biomedicine Nanchang China; ^2^ Department of Ophthalmology The First Affiliated Hospital of Nanchang University, Jiangxi Branch of National Clinical Research Center for Ocular Disease Nanchang China; ^3^ Department of Ophthalmology and Visual Sciences The Chinese University of Hong Kong Hong Kong China; ^4^ Department of Ophthalmology, Massachusetts Eye and Ear Harvard Medical School Boston Massachusetts USA; ^5^ School of optometry and vision science Cardiff University Cardiff UK

**Keywords:** fALFF, functional MRI, inner brain activity, toothache

## Abstract

**Background:**

Previous studies have indicated that pain‐related diseases can result in significant functional alterations in the brain. However, differences in spontaneous brain activity in toothache (TA) patients remain unclear.

**Objective:**

To investigate altered spontaneous brain activity in patients with TA and its underlying mechanisms using the resting‐state functional magnetic resonance imaging–fractional amplitude of low‐frequency fluctuation (rsfMRI–fALFF) technique.

**Methods:**

Twelve patients with TA and 12 non‐toothache controls (NTCs) (matched for sex, age, and level of education) were enrolled. Spontaneous cerebral activity variations were investigated using the rsfMRI–fALFF technique in all individuals. The mean fALFF values of the TA patients and NTCs were classified using receiver operating characteristic (ROC) curves. The correlations between fALFF signals of distinct brain regions and clinical manifestations of TA patients were evaluated using Pearson's correlation analysis.

**Results:**

TA patients showed lower fALFF values in the left superior frontal gyrus, medial; right superior frontal gyrus, dorsolateral; and left median cingulate and paracingulate gyri (LDCG) than the NTCs. Moreover, ROC curve analysis indicated that the area under the curve of each cerebral region studied had high accuracy. Besides, in the TA group, the visual analog scale score was negatively correlated with fALFF signal values of the LDCG (*r* = .962, *p* < .001).

**Conclusion:**

Abnormal spontaneous activity was detected in numerous brain regions in patients with TA, which may be valuable for understanding the brain processing mechanism underlying TA. These regional changes in brain activity may serve as effective clinical indicators of TA.

## Graphical abstract

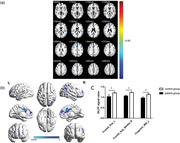

## 1 INTRODUCTION

Any oral disease may cause toothache (TA). In addition to tooth disease, periodontal tissue and jaw diseases and various nerve and systemic diseases can also lead to TA (Raab, 1991). The prevalence estimates for dental pain have been reported ranging from 7% to 32% for “TA” and 7% to 66% for overall dental pain (Pau et al., 2003). Thus, this condition is regarded as a public health problem. Understanding the mechanism of TA could contribute to not only treatment but also prevention of its development. However, regardless of the factors that induce TA (except for the different excitatory mechanisms of the peripheral nerve), TA typically involves the introduction of a harmful stimulus and processing by the central nervous system to manage and regulate afferent information.

The formation of TA is a complex process that is related not only to craniofacial sensory neurophysiology but also to emotional, pain behavioral, cognitive, and psychological factors (Tracey & Mantyh, 2007). At present, understandings of the changes of corresponding cerebral activities of TA and the underlying neuropathological mechanism are limited. Previous study preliminarily elucidated the role of the caudal anterior cingulate cortex (ACC) in the regulation of TA‐related affective behaviors using positron emission tomography (Yu et al., 2022). However, numerous aspects of the brain processing mechanism of TA and its impact on cognitive function remain indistinct. Therefore, to deal with TA more effectively, understanding the brain mechanisms that shape subjective pain experiences is critical.

Functional magnetic resonance imaging (fMRI) is one of the most widely used tools for studying the neural substrates of the pain experience (Lin et al., 2014; Pan et al., 2018). This technique allows researchers to observe changes in neural activity in specific brain regions (Brown et al., 2016; Goodyear & Menon, 2001). Previous reports have used fMRI to confirm that various experimental nociceptive stimuli activate several brain regions, which include the primary somatosensory cortex (S1), secondary somatosensory cortex (S2), thalamus, ACC, and insula (Apkarian et al., 2005; Dasilva et al., 2002; Fomberstein et al., 2013). Moreover, previous studies have indicated that electrical stimulation of the teeth increases the blood oxygen level‐dependent (BOLD) signal in the cerebrum. And fMRI can also be used to study the spontaneous neural activity of patients. For instance, patients with trigeminal neuralgia exhibited BOLD activation in specific cerebral regions such as the somatosensory cortex (Moissetl et al., 2011). Amplitude of low‐frequency fluctuation (ALFF) is a resting‐state fMRI (rs‐fMRI) analysis technique that reflects the spontaneous activity of neurons, and changes in ALFF are positively correlated with changes in BOLD signal intensity in certain brain regions (Tan et al., 2016). In other words, a higher ALFF value indicates greater active neural activity. Fractional ALFF (fALFF) approach, in which the ratio of power spectrum of low‐frequency (0.01−0.08 Hz) range to that of the entire frequency range was computed, effectively suppresses nonspecific signal components in the rs‐fMRI, and therefore would significantly improve the sensitivity and specificity in detecting regional spontaneous brain activity (Zou et al., 2008). Besides, the application of fALFF in psychiatric diseases is widespread. For instance, fALFF values can show the effects of cognitive therapy and antidepressants on the brain activity of patients with depression and reflect the efficacy (Shu et al., 2020).

fALFF can serve as a new indicator of the level of TA. As pain experience is subjective, it is crucial to adopt more objective methods to measure one's pain level, which contribute to the diagnosis of illness and the following treatment.

Therefore, fALFF is a reliable fMRI technology that has not been applied in TA yet. fALFF values were used in this study to compare spontaneous brain activity between TA patients and non‐toothache controls (NTCs).

## 2 MATERIALS AND METHODS

### 2.1 Subjects

In this study, a total of 12 TA patients (*n* = 4 males and *n* = 8 females) were recruited from the Affiliated Stomatological Hospital of Nanchang University and the First Affiliated Hospital of Nanchang University. Inclusion criteria of TA patients were as follows: (1) pain in the pulp or periodontal tissue of teeth of either dental or nondental origin; (2) acute and chronic TA; (3) no other pain diseases; (4) capable of undergoing MRI examination; (5) no abnormal signal changes observed during conventional MRI examination; (6) no history of drug and alcohol addiction; and (6) TA has no obvious cause and cannot be attributed to any other diseases. Exclusion criteria were as follows: (1) headache, temporomandibular disorders, fibromyalgia, back pain, or other non‐TA conditions; (2) first‐generation family history of pain, such as headaches; (3) other neurological or psychiatric disorder; and (4) contraindications to MRI.

Twelve NTCs matched for sex, age, and educational status were also recruited from the Department of Ophthalmology, the First Affiliated Hospital of Nanchang University, comprising four males and eight females. Inclusion criteria of NTCs were as follows: (1) no symptoms of TA; (2) no under obvious pain conditions; (3) no abnormalities of cerebral parenchyma on the MRI examination; (4) no cardiovascular disease, mental illness, or cerebral infarction; (5) no history of drug and alcohol addiction; (6) no contraindications for MRI examination, such as implanted metal devices or a cardiac pacemaker; and (7) can tolerate MRI examination.

This study complied with the Declaration of Helsinki and was formally approved by the Medical Ethics Committee of the Affiliated Stomatological Hospital and the First Affiliated Hospital of Nanchang University. The Medical Ethics Committee also approved the study. All volunteers provided written informed consent after they were informed of the study objectives, protocol, and inherent risks of the study.

### 2.2 Pain scores

The visual analog scale (VAS) was used to measure pain in TA patients. Using a 10‐cm ruler, patients ranked the degree of pain they were currently experiencing on a scale of 0–10. A higher score indicated more intense pain. Patients rated their pain on the VAS just before the scanning session. NTCs rated on the same time while their pain did not refer to a specific kind of pain.

### 2.3 MRI data acquisition

All individuals underwent conventional MRI before BOLD cerebral function examination using rs‐fMRI, and no abnormal signal changes in cerebral structure were observed. The MRI scanning was performed using a 3T magnetic resonance scanner with an eight‐channel phased‐array head coil (Trio, Siemens, Munich, Germany). The TA group underwent rs‐fMRI scanning under the condition of persistent TA. During the entire scanning process, all participants were instructed to remain awake while breathing regularly with their eyes closed. The rs‐fMRI data were acquired using a gradient echo‐echo planar imaging sequence. Relevant detailed parameters were as follows: 30 axial slices and 240 functional images (gap: 1 mm; repetition time [TR]: 2000 ms; echo time [TE]: 40 ms; flip angle: 90°; field of view [FOV]: 240 × 240 mm; thickness: 4.0 mm; in‐plane resolution: 64 × 64). In addition, we obtained structural images for each individual using a T1‐weighted three‐dimensional magnetization‐prepared 180 degrees radiofrequency pulses and rapid gradient‐echo sequence (176 structural images; acquisition matrix: 256 × 256; TR: 1900 ms; TE: 2.26 ms; flip angle: 9°; FOV: 240 × 240 mm; thickness: 1.0 mm). The entire scanning process lasted 15 min.

### 2.4 fMRI data processing

No filtering was used in the preprocessing. We first applied the MRIcro software (MRIcro 1.4, MRIcro software guide [sc.edu]) to remove incomplete data. During magnetization equilibration, the first 15 time points were discarded. The Data Processing Assistant for Rs‐fMRI Advanced Edition (DPARSFA 4.0; http://rfmri.org/DPARSF) software was used for head motion correction, spatial normalization, slice timing, digital imaging communications in medicine form transformation, and full‐width smoothing with a Gaussian kernel of 6 × 6 × 6 mm^3^ at half‐maximum, based on the rs‐fMRI data analysis toolkit (REST; http://www.restfmri.net) and Statistical Parametric Mapping software (SPM8; http://www.fil.ion.ucl.ac.uk/spm). We would have excluded subjects if they have had excessive angular motion or >1.5 mm maximum shift in the *x*, *y*, or *z* direction during the fMRI examination. No participants were excluded due to head motion.

M 199 False variables with signals from a region centered on the brain white matter and a ventricular region of interest (ROI) were eliminated using linear regression. After correcting for head motion, the functional images were normalized to Montreal Neurological Institute space using a standard echo‐planar image template. The global effects of variability were reduced by dividing the fALFF value of each voxel by the global mean fALFF value of each subject. Hz range within which fALFF of each voxel was calculated.

### 2.5 Brain–behavior correlation analysis

The REST software was used to classify brain regions with differences in fALFF value between the two groups as ROIs. The mean fALFF value was calculated for each ROI by averaging across all voxels within the ROI. The relationship between behavioral performance and the mean fALFF value of each ROI was analyzed using linear correlation analysis in the TA group. A *p* < .05 was considered statistically significant.

### 2.6 Statistical analysis

The SPSS software version 26.0 was used to compare demographic and behavioral variables between the TA and NTC groups using independent sample *t*‐tests and chi‐square tests. Differences with a *p* < .05 were considered statistically significant. A two‐sample *t*‐test was used to compare functional data using the REST software. The voxel‐level statistical threshold for multiple comparisons using Gaussian random field theory was set at *p* < .05. The voxel‐level AlphaSim was corrected at *p* < .01 with a cluster size of >40 voxels. Receiver operating characteristic (ROC) curves were used to categorize brain regions that significantly differed in mean fALFF values between TA subjects and NTCs. The correlations between the obtained fALFF values and clinical features of TA patients were investigated using Pearson's correlation analysis.

## 3 RESULTS

### 3.1 Characteristics of the study participants

Age did not significantly differ between the TA and NTC groups (*p* = .704). The educational level of all patients matched those of the NTCs. The duration of TA was 1.92 ± 0.76 years. Table 1 describes the details of the study participants.

**TABLE 1:** Demographic and behavioral comparisons between the TA and HC groups

	TA	HC	*t*‐value	*p*‐value
Male/female	4/8	4/8	N/A	>.99
Age (years)	42.26 ± 9.65	40.19 ± 12.64	0.081	.704
Handedness	12R	12R	N/A	>.99
Duration (months)	1.92 ± 0.76	N/A	N/A	N/A
VAS	6.67 ± 2.39	N/A	N/A	N/A

*Note*: Independent *t*‐tests comparing the two groups (significant at *p* < .05). Data are presented as means ± standard deviations.

Abbreviations: HC, healthy control; N/A, not applicable; R, right; TA, toothache; VAS, visual analog scale.

### 3.2 fALFF differences

**FIGURE 1:**
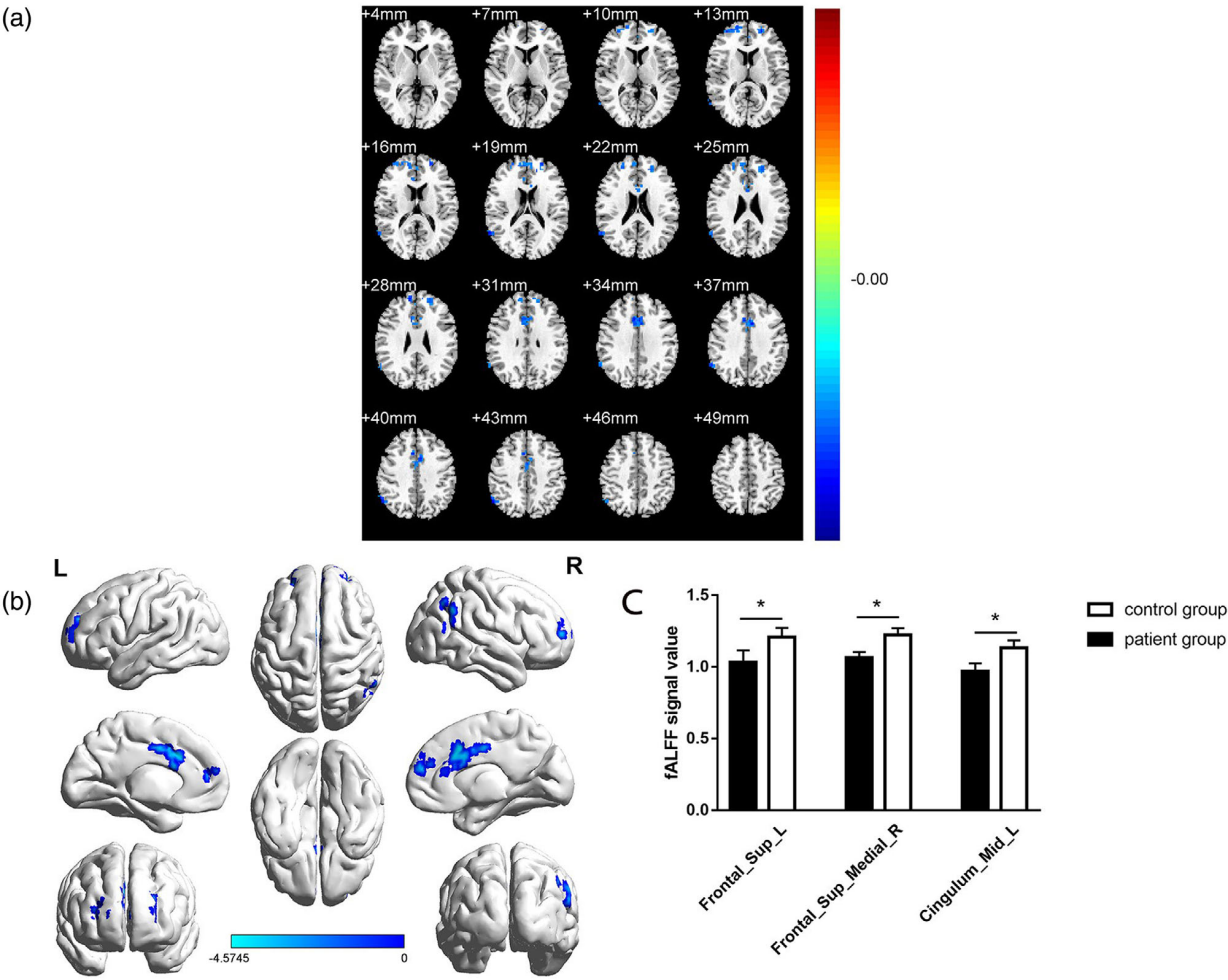
Spontaneous brain activity in TA patients and HCs. (A, B) Regions with significant group differences in brain activity. Blue regions indicate lower fALFF values (AlphaSim corrected at a cluster size of >40 voxels and *p* < .05). (C) Mean fALFF values of the TA patients and HCs. fALFF, fractional amplitude of low‐frequency fluctuation; TA, toothache; HC, healthy control.

The fALFF values of the TA group were lower than NTCs in several brain regions: left superior frontal gyrus, medial (LSFGmed), right superior frontal gyrus, dorsolateral (RSFGdor), as well as the left median cingulate and paracingulate gyri (LDCG) (Figure 1a,b; Table 2). Mean fALFF values of the two groups are shown in Figure 1c.

**TABLE 2:** Brain regions with significant differences in fALFF between TA patients and HCs

L/R	Brain regions	BA	MNI coordinate			Number of voxels	*t*‐value
			*X*	*Y*	*Z*		
L	Superior frontal gyrus, dorsolateral	3	−21	57	12	72	−4.3742
R	Superior frontal gyrus, medial	24	12	54	27	113	−4.5032
L	Median cingulate and paracingulate gyri	33	−6	12	36	159	−4.4368

*Note*. The statistical threshold was set at a voxel level of *p* < .01 for multiple comparisons.

Abbreviations: BA, Brodmann area; fALFF, fractional amplitude of low‐frequency fluctuation; HC, healthy control; L, left; MNI, Montreal Neurological Institute; R, right; TA, toothache.

### 3.3 ROC curve

**FIGURE 2:**
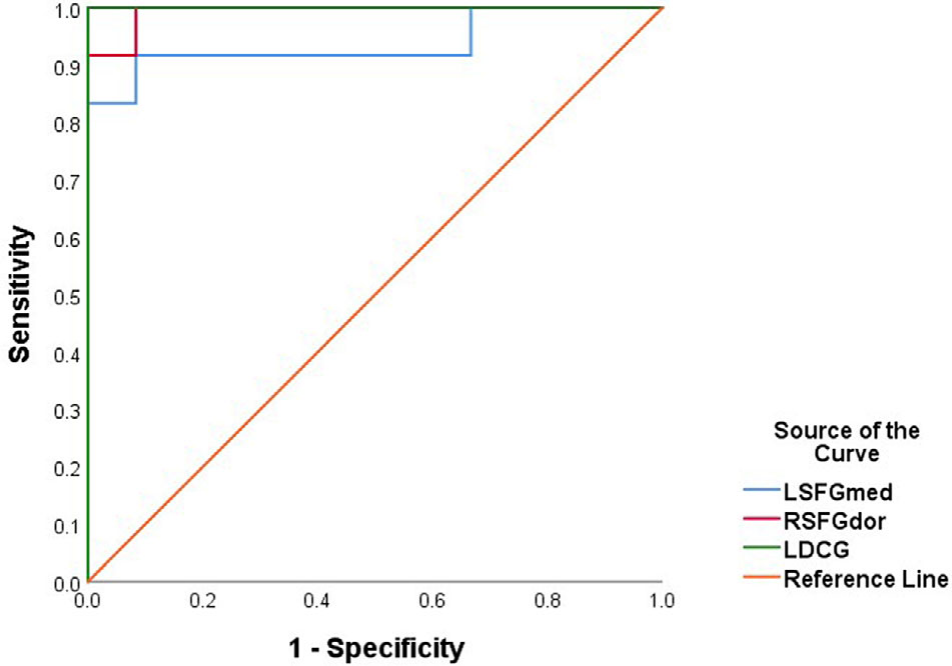
ROC curve analysis of the mean fALFF values for distinct brain activity regions. The AUC was .937 for LSFGmed (*p* < .0001; 95% CI: .827−1.000; SE = .056). Specificity was .993 for RSFGdor (*p* < .0001; 95% CI: .971−1.000; SE = .011) and 1.000 for LDCG (*p* < .0001; 95% CI: 1.000−1.000; SE = .001). AUC, area under the curve; ROC, receiver operating characteristic; LSFGmed, left superior frontal gyrus, medial; RSFGdor, right superior frontal gyrus, dorsolateral; LDCG, left median cingulate and paracingulate gyri; SE, standard error.

We predicted that fALFF values would be useful diagnostic markers for distinguishing TA patients from NTCs. This was tested using the ROC curve method. We calculated the mean fALFF values of the distinct brain areas between TA patients and NTCs. Accuracy was considered low if the area under the curve (AUC) was .5−.7, whereas an AUC of .85−.95 denoted high accuracy. Individual AUC values for the fALFF value of different regions were as follows: LSFGmed, .937 (*p* < .0001; 95% confidence interval [CI]: .827−1.000); RSFGdor, .993 (*p* < .0001; 95% CI: .971−1.000); LDCG, 1.000 (*p* < .0001; 95% CI: 1.000−1.000) (Figure 2).

### 3.4 Correlation analysis

**FIGURE 3:**
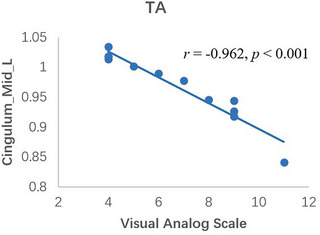
Correlations between the fALFF values of different regions and the clinical behaviors in the TA group. (A) The VAS negatively correlated with fALFF signal values of the LDCG (*r* = .962, *p* < .001). (B) There is no correlation between the VAS and the LDCG in HCs. fALFF, fractional amplitude of low‐frequency fluctuation; TA, toothache; VAS, visual analog scale; LDCG, left median cingulate and paracingulate gyri.

The result of the linear correlation analysis showed that in the TA group, the fALFF values in the LDCG were negatively correlated with the VAS score (*r* = −.962, *p* < .001; Figure 3), while neither the LSFGmed nor the RSFGdor has significant correlation between fALFF values and the VAS score.

## 4 DISCUSSION

fMRI, which is based on the blood oxygen level, has high spatial resolution, which provides a crucial means for studying cerebral function (Ogawa et al., 1990). In particular, rs‐fMRI has been used widely in the field of neuroscience and neuropsychiatric diseases because it reflects the spontaneous neural activity of the human brain (Biswal et al., 1995). fALFF divides the energy of the calculated low‐frequency signal by the power of the whole frequency band, which effectively addresses the disadvantages of ALFF and improves the sensitivity and specificity of spontaneous neural activity signal detection (Hu et al., 2022). Moreover, the fALFF method has successfully been applied to several pain‐related diseases and is expected to have wide prospects (Table 3) (Dai et al., 2020; Li et al., 2021; Schneider et al., 2020; Wang et al., 2016; Zhang et al., 2019, 2021).

**TABLE 3:** fALFF method applied in pain‐related diseases

Authors	Years	Diseases
Wang et al.	2016	Migraine
Zhang et al.	2019	Trigeminal neuralgia
Dai et al.	2020	Postherpetic neuralgia
Schneider et al.	2020	Chronic low back pain
Zhang et al.	2021	Neovascular glaucoma
Li et al.	2021	Normal‐tension glaucoma

**FIGURE 4:**
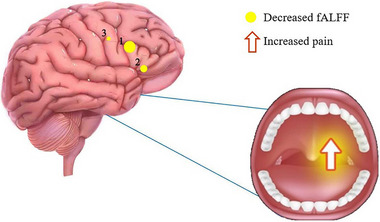
The mean fALFF values of altered brain regions. The fALFF values were decreased to various extents compared with the HCs in the following regions: 3‐LSFG (BA 3, *t* = −4.3742), 1‐RSFG (BA 24, *t* = −4.5032), and 2‐LDCG (BA 33, *t* = −4.4368). The sizes of the spots denote the degree of quantitative changes. HCs, healthy controls; BA, Brodmann's area; LSFGmed, left superior frontal gyrus, medial; RSFGdor, right superior frontal gyrus, dorsolateral; LDCG, left median cingulate and paracingulate gyri.

**FIGURE 5:**
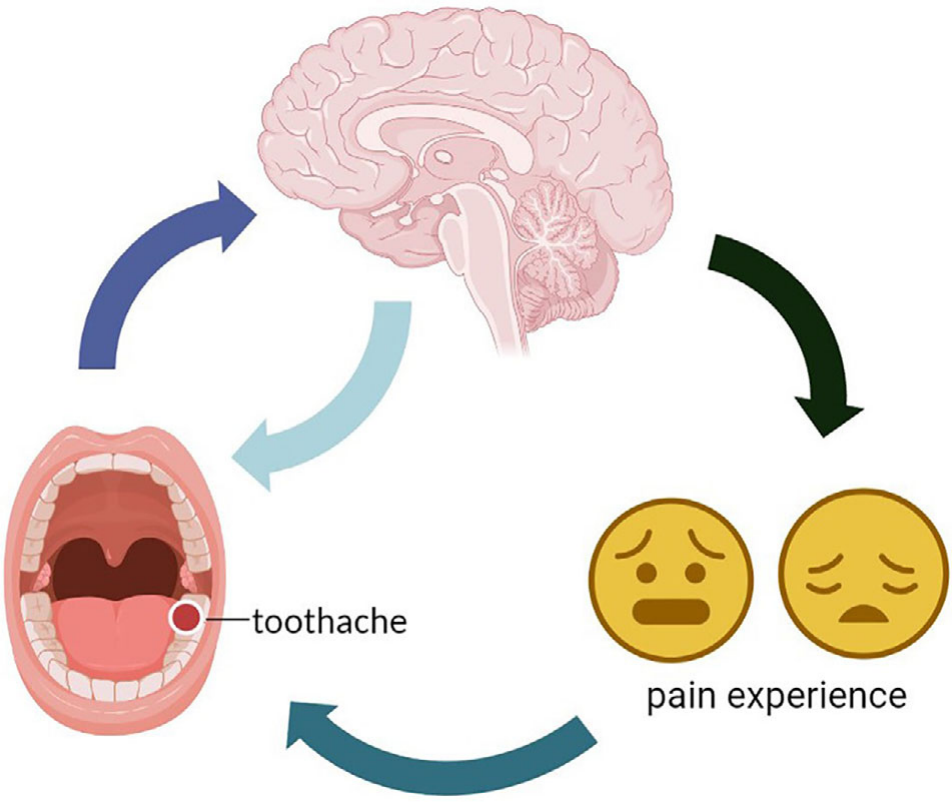
Relationship between fALFF values and the experience of pain. The mean fALFF values presented obvious abnormalities in many specific cerebral regions of TA patients in contrast to HCs, and TA patients appear to be more prone to feel painful. A schematic diagram of the relationship between the effects of toothache on mood and changes in neural activity in related cerebral regions.

In this study, we demonstrated that the patterns of activity in different brain regions were altered in TA patients compared with NTCs. fALFF values of TA patients were lower in the RSFGmed, LSFGdor, and LDCG (Figure 4). The TA group in this study showed significant correlations between fALFF values in cerebral regions and VAS scores, indicating that pain index is linked with fALFF values, and that abnormal neural electrical activity may occur in brain regions associated with the experience of pain (Figure 5). We did not find any regions of the brain with higher fALFF values in TA patients compared with NTCs; therefore, this is not discussed in this article.

The complete experience of pain includes sensory, emotional, and cognitive factors (Tracey & Mantyh, 2007). The sensory pathway of pain is composed of the lateral nucleus of the thalamus and its corresponding cortical projection area, the S1 and S2, and is called the lateral pain system (Al‐Chalabi et al., 2022). The emotional pathway of pain is composed of the prefrontal lobe, the medial nucleus of the thalamus, and the limbic system that these regions project to and is known as the medial nociceptive system (Ossipov et al., 2010).

Both the qualitative and quantitative analyses have demonstrated activation of the somatosensory cortex, insula, and cingulate cortex under experimental “TA” evoked using pulpal electrical stimulation, which suggests that dental pain is associated with the core pain‐related network (Lin et al., 2014). However, these activated areas may not be fixed; rather, they may vary across individuals and sources of pain. Furthermore, an fMRI study on dentoalveolar dynamic pressure pain in subjects without pain revealed activation of brain areas typically associated with pain processing, such as the thalamus, S1, S2, insula, and prefrontal cortex (Moana‐Filho et al., 2010). Moreover, dental pain has been shown to be associated with the cognitive–affective network related to pain (Lin et al., 2014).

The superior frontal gyrus (SFG) is involved in motor activity (Martino et al., 2011), working memory (Rowe et al., 2000), and resting‐state regulation (Briggs et al., 2020) and is considered a core brain region of the cognitive control system (Niendam et al., 2012). The function of the SFG has also been shown to be associated with emotion regulation‐related processes (Frank et al., 2014). A whole‐brain correlation analysis study demonstrated that fALFF values increase in the left SFG when perceived stress levels are higher (Wang et al., 2019). As the formation of TA is influenced by cognitive and emotional factors (Tracey & Mantyh, 2007), as well as stress (Giannakopoulos et al., 2010; Yang et al., 2016), SFG might play a vital role in the regulation of TA. Compared with NTCs, the percent amplitude of fluctuation signals in patients with Moyamoya disease is lower in the RSFGmed (Li et al., 2021). Therefore, the mechanism of fALFF value decrease in this brain area induced by TA may be the same as that caused by Moyamoya disease. However, whether and how TA results in a decreased blood supply to this region remain unknown. Additionally, researchers have found that weak tooth stimulation induces greater BOLD changes in the medial frontal and right superior frontal gyri than that induced by strong tooth stimulation (Jantsch et al., 2005). This may indicate that BOLD signal intensity is affected by the degree of pain. Therefore, our study has certain limitations because the results may be influenced by different intensities of pain patients experienced, which were measured by the completely subjective VAS score.

The cingulate cortex is divided into the ACC, midcingulate cortex, and posterior cingulate cortex (PCC). The ACC is involved in emotion and movement, whereas the PCC is mainly engaged in memory and some visual functions (Vogt et al., 1992). Devinsky et al. (1995) further subdivided the ACC into anterior and posterior sections, where the function of the anterior ACC is generating emotions and autonomous responses, and the posterior ACC houses the motor and pain‐related areas of the ACC. As part of the limbic system, the cingulate gyrus is one of the components of the medial pain system, and the processing of pain information is primarily performed by the ACC. Moreover, functional imaging investigations have indicated that the ACC mediates affective responses to noxious stimuli (Reiner, 1990). However, only part of the ACC is involved in emotion, and the various sections of the ACC contribute variably to the affective responses associated with pain (Vogt, 2005).

Electrical dental stimulation elicited neuronal activation in clusters covering the anterior midcingulate cortex (aMCC) and posterior midcingulate cortex and, to a lesser extent, the pregenual ACC (pgACC). Among these, the contralateral (left) aMCC and pgACC showed linear BOLD signal changes that correlated to physical stimulus strength. Generally, the aMCC divisions are associated with cognitive–evaluative functions, whereas the pgACC is linked to emotional and/or affective dimensions of stimulus perception as well as to skeletomotor orientation in response to pain (Brügger et al., 2012). We previously found that mild cognitive impairment (MCI) and pre‐MCI groups show common abnormalities of dynamic functional connectivity in the left middle temporal gyrus, LDCG, and left thalamus (Wang et al., 2022). Furthermore, to summarize the above discussion, we have provided a summary of the functions of the abovementioned brain regions and the effects of corresponding dysfunctions in Table 4.

**TABLE 4:** Brain regions alteration and its potential impact

Brain regions	Experimental result	Brain function
Superior frontal gyrus, dorsolateral	TAs < HCs	Working memory, divided attention, cognitive control
Superior frontal gyrus, medial	TAs < HCs	Conflict monitoring, error detection, attention control
Cingulate gyrus	TAs < HCs	Pain formation, cognitive control, emotion processing

We found that the fALFF values of the LDCG were strongly negatively correlated with the VAS score (*r* = −.962, *p* < .001), which indicated that, as the degree of TA increased, the BOLD signal intensity of this region declined. This may be related to the reduction in blood supply or neuronal activity in this region. It is known that the cingulate gyrus is involved in the formation of pain sensation and the encoding of pain emotional information (Zhuo, 2007); thus, there may be changes in neuronal activity or hemodynamics of the cingulate gyrus during executive processes, although the specific mechanisms remain uncertain. This finding not only reflects the close relationship between the cingulate gyrus and pain sensation but also offers promise for fALFF values of the LDCG to be used as an indicator of the level of TA. Thus, our study has yielded a novel finding and provides new avenues for further research.

This study has several limitations. First, our sample size was small. Studies in larger sample sizes are needed to obtain more precise results. And classification with the ROC curve done on such a small sample size is a statistical limitation. Second, the inclusion criteria were not rigorous; acute and chronic TAs were not differentiated. Third, medication usage, which might affect brain activity, was not included in the exclusion criteria. In addition, we did not classify odontogenic and nonodontogenic pain in the inclusion criteria for patients with TA. It is also important to note that, even under the same conditions, the pain experience is not static over time within an individual; moreover, pain perception fluctuates over time (Bi et al., 2021). Besides, the exact functions of the brain regions involved in this study are unclear; therefore, the specific reasons for the decline in fALFF value in these brain regions induced by TA cannot be fully explained. The aim of our study was not mechanistic and could at best inform future longitudinal and intervention studies that are needed to discern mechanisms. In addition, differences between acute and chronic pain in relation to fALFF should be investigated. Including depression and anxiety as covariates since they are relevant for brain changes and develop commonly in chronic pain states could be a recommendation for future studies as well.

Taken together, we revealed that patients with TA have abnormal spontaneous neural activity in specific brain regions, which provided insight into the variation of central nervous system activity in TA patients and potential pathological mechanisms underlying TA pain. In addition, we revealed a strong correlation between the VAS score and fALFF value of the LDCG, which suggested that the degree of pain is closely related to the cingulate gyrus. At present, there are few studies on the reductions in BOLD signal intensity in the brain and the mechanisms underlying painful conditions. Our study provides new research avenues for investigating the relationship between various painful conditions and BOLD signal intensity in specific brain regions. Moreover, our findings offer support for the use of fALFF values as an indicator of pain degree. Since fALFF reflects changes in spontaneous brain activity and is entirely objective, it is conducive to our evaluation of pain intensity and the state of illness.

## 5 CONCLUSION

This study showed that patients with TA have abnormal spontaneous neural activity in specific brain regions and that fALFF values of the LDCG are strongly correlated with the VAS score. Our findings provide insight into the variation of central nervous system activity in TA patients and indicate potential pathological mechanisms underlying TA pain. We suggest that the cingulate gyrus plays a vital role in the production and formation of TA and that fALFF signals could be an effective marker of pain severity.

## CONFLICT OF INTEREST STATEMENT

The authors declare no conflicts of interest.

### PEER REVIEW

The peer review history for this article is available at https://publons.com/publon/10.1002/brb3.2937.

## Data Availability

The datasets used and/or analyzed during the present study are available from the corresponding author on reasonable request.
